# Targeting the Androgen Receptor and Associated Cofactors in Prostate Cancer: Novel Approaches and Future Perspectives

**DOI:** 10.3390/ijms27188200

**Published:** 2026-09-15

**Authors:** Paulina J. Dziubańska-Kusibab, Thibaud Jourdan, Bernard Haendler

**Affiliations:** Research and Early Development Oncology, Pharmaceuticals, Bayer AG, Müllerstr. 178, 13353 Berlin, Germany; paulina.dziubanska-kusibab@bayer.com (P.J.D.-K.); thibaud.jourdan@bayer.com (T.J.)

**Keywords:** prostate cancer, androgen signaling, degradation, splicing, pioneer factor, chromatin modulation, nanoparticle, biomolecular condensate

## Abstract

Following the first approval for prostate cancer (PCa) treatment of competitive antagonists of the androgen receptor (AR) function that bind to the androgen-binding pocket, much progress has been achieved regarding compound efficacy and specificity. Second-generation compounds and androgen synthesis blockers are now standard-of-care AR pathway inhibitors given to PCa patients. More recently, approaches targeting AR regions other than the ligand-binding domain (LBD), stimulating proteasome-mediated AR degradation, or bringing together the AR with an effector protein (EP) in prostate tumor cells, have been explored and, in several cases, clinically tested. Compounds blocking AR mRNA splicing or directly addressing AR splice variants, especially the constitutively active AR-V7 form, are also being evaluated. In addition, there are ongoing efforts aiming at impairing the function of essential AR cofactors, mainly those involved in downstream gene transcription and chromatin modulation. Alternative techniques to impair AR activity, such as nanoparticle formulations and targeting biomolecular condensates are furthermore being explored. Here we present the most recent developments in these different strategies to target the AR, directly or indirectly, for a more potent and long-lasting blockade. This will hopefully soon lead to treatments that improve progression-free and overall survival in PCa patients, while maintaining a good safety profile.

## 1. Introduction

Prostate cancer (PCa) is the second most frequently detected tumor in men worldwide [1], and will represent about 31% of new cancer cases and account for 11% of cancer-related deaths in the USA in 2026 [2]. Localized PCa has a relative 5-year survival of 97% [2] and is usually managed with active surveillance, surgery or local radiation [3]. Once the disease progresses, androgen deprivation therapy (ADT) with gonadotropin-releasing hormone (GnRH) antagonists or agonists, and androgen receptor (AR) signaling inhibitors directly targeting the AR or blocking the androgen synthesis pathway represent the treatments of choice [3,4]. The AR is a ligand-dependent transcription factor (TF) that will bind to specific androgen response elements (AREs) in the cell nucleus following stimulation by the androgen dihydrotestosterone (DHT) while interacting with different cofactor proteins, thus leading to downstream regulation of target genes [5,6].

The first AR-targeting compounds, flutamide, nilutamide and bicalutamide, were approved over 30 years ago, later followed by second-generation AR inhibitors including darolutamide, enzalutamide, apalutamide and rezvilutamide, and the CYP17A1 inhibitor abiraterone acetate which blocks androgen biosynthesis [4,7,8]. These second-generation compounds show high efficacy across PCa disease states (Table 1) with good safety profiles [9,10]. Unfortunately, in most patients, the tumor will adapt to the therapy and ultimately relapse [4,11]. This is often linked to elevated AR expression due to gene amplification or enhancer hijacking, to emergence of AR splice variants, to point mutations in the androgen-binding pocket, and also to aberrant AR cofactor activities [6,12,13,14,15,16,17,18,19]. Subsequent treatments such as chemotherapy with the taxanes docetaxel or cabazitaxel, and more recently, radioligand therapy with radium-223 dichloride or lutetium-177-PSMA-617, and poly(ADP-ribose) polymerase (PARP) inhibitors have been approved [3,20,21]; however, the median survival rate for late-stage, metastatic castration-resistant prostate cancer (mCRPC) remains below 3 years [22]. Neuroendocrine prostate cancer (NEPC), which is diagnosed in less than 1% of de novo PCa cases, has an increased incidence in late-stage disease, currently amounting to 15–25%, with only a seven-month-long median survival time in post-ADT mCRPC patients [23,24]. Different NEPC subtypes with normal, reduced or undetectable AR expression levels and neuroendocrine-positive or -negative status have been described [25,26]. The AR-negative/NE-negative subtype has the lowest survival expectancy and carries unique genetic and epigenetic features [25,26].

**Table 1:** Overview of selected approved and clinically advanced compounds for PCa treatment. Combination partners used for co-treatment are not listed.

Strategy	Representative Agent	Target	Disease Setting	Development Stage	Comments
Competitive AR antagonist	Enzalutamide	AR LBD	mCRPC; nmCRPC; nmHSPC	Approved	Additional clinical studies ongoing
	Apalutamide	AR LBD	nmCRPC; mHSPC	Approved	
	Darolutamide	AR LBD	nmCRPC; mHSPC	Approved	
SARD	ONCT-534	AR	mCRPC	Phase 1/2 (NCT05917470)	Terminated
AR PROTAC	Gridegalutamide	AR LBD	mCRPC	Phase 3 (NCT06764485)	Ongoing
	Luxdegalutamide	AR LBD	mHSPC	Phase 2 (NCT06991556)	Ongoing
			mCRPC	Phase 2 (NCT07047118)	Ongoing
	Bavdegalutamide	AR LBD	mCRPC	Phase 1/2 (NCT03888612)	Completed
AR RIPTAC	HLD-0915	AR	mCRPC	Phase 1/2A/2B (NCT06800313)	Ongoing
AR-V7 inhibitor	Masofaniten	AR NTD	mHSPC	Phase 2 (NCT06312670)	Completed
			mCRPC	Phase 2 (NCT05075577)	Terminated
AR splicing	EZN-4176	AR exon 4	CRPC	Phase 1a/1b (NCT01337518)	Suspended
Inhibition of epigenetic player	Mevrometostat	EZH2	mHSPC	Phase 3 (NCT07028853)	Ongoing
			mCRPC	Phase 3 (NCT06629779)	Ongoing
	KTX-2001	NSD2	mCRPC	Phase 1 (NCT07103018)	Ongoing
	Inobrodib	CBP/p300	mCRPC	Phase 1/2a (NCT03568656)	Completed
	Pocenbrodib	CBP/p300	mCRPC	Phase 1b/2a (NCT06785636)	Ongoing
	EP31670	CBP/p300 + BET	CRPC	Phase 1 (NCT05488548)	Ongoing
	ZEN-3694	BET	mCRPC	Phase 2 (NCT04471974)	Ongoing

Detailed examinations of biopsies from normal and cancerous prostate using innovative approaches such as single-cell transcriptomics and spatial analyses have unraveled the complexity of primary and castration-resistant tumors, and heterogeneous epithelial and microenvironmental cell states likely to be involved in future spread have been identified [27,28,29,30,31]. Tumor-associated precursor cells with different epithelial identities play a crucial role in cancer progression, as reported by several groups [27,31,32,33,34,35]. The sharp rise in data originating from multi-omics approaches has also led to crucial discoveries related to the response to established and exploratory treatments [30,36,37].

The transcriptional switch between the normal function of the AR in maintaining prostate physiology towards a tumor-promoting activity in early- and late-stage PCa is gradually being unraveled [38,39]. Recent studies additionally revealed the role of local nuclear AR condensates in the regulation of transcription programs with oncogenic potential [40,41]. Importantly, despite substantial advances in our understanding of the complexity of PCa biology, the AR remains an essential player in PCa and innovative strategies to block its activity directly or indirectly are intensively being evaluated.

**Figure 1:**
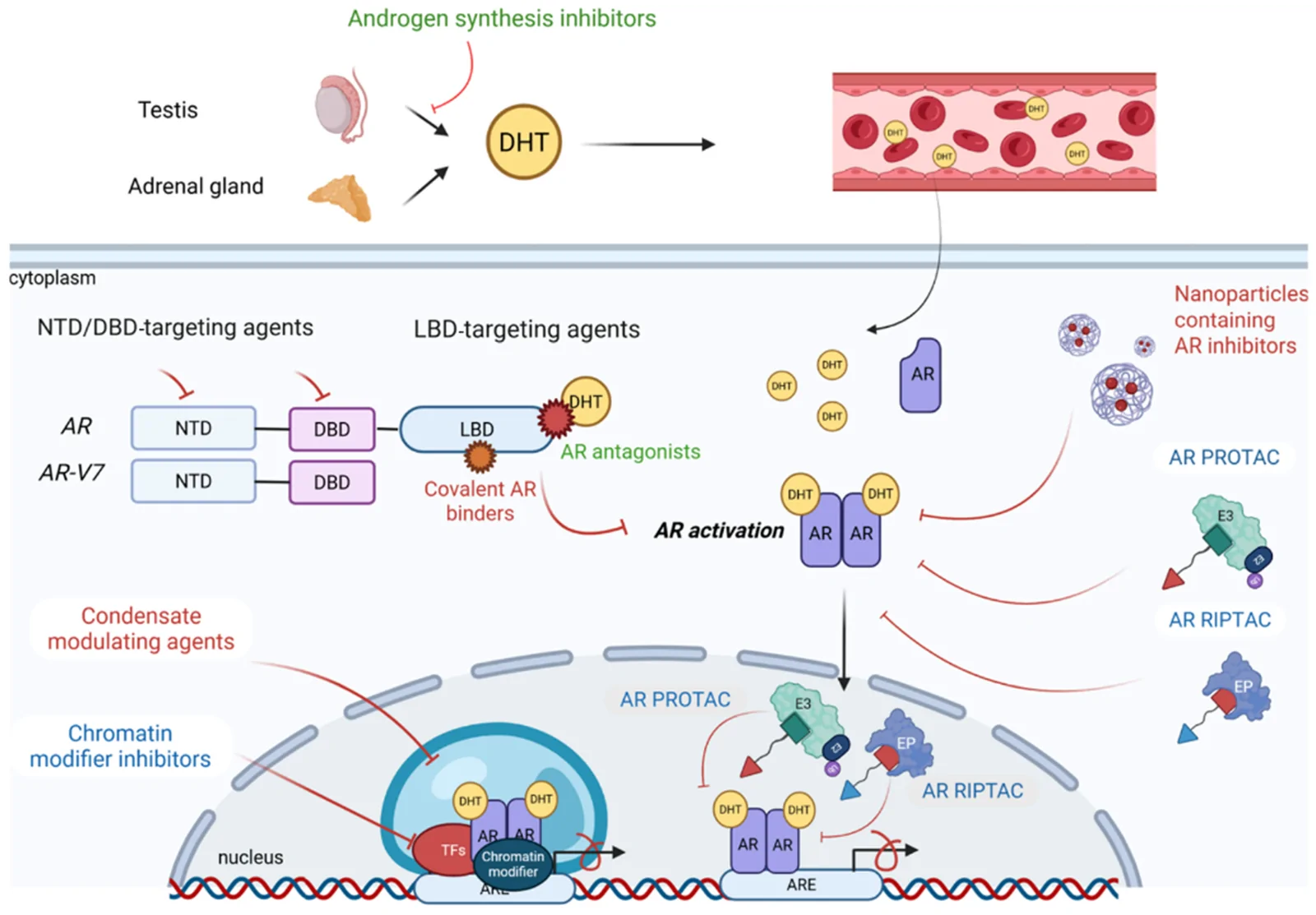
Schematic overview of selected approaches to target the AR and associated cofactors in PCa. Approved therapies are indicated in green, clinical approaches in blue and early studies in red. Created in BioRender: Jourdan, T. (2026) https://BioRender.com/u7jm4eu (accessed on 21 August 2026).

As mentioned, several inhibitors directly competing with androgens for binding to the AR ligand-binding domain (LBD) are approved for PCa treatment. New approaches to target this domain with selective AR degraders (SARDs), or with novel modalities such as Proteolysis-Targeting Chimeras (PROTACs) or Regulated Induced Proximity Targeting Chimeras (RIPTACs), are now being explored and tested. Also, agents addressing other AR regions in the N-terminal domain (NTD) or the DNA-binding domain (DBD), which are maintained in essential splice variants, are being investigated. They include compounds that prevent AR dimerization or inhibit interactions with essential cofactors or with DNA. Efforts are moreover ongoing to better understand the mechanisms responsible for aberrant splicing of AR transcripts and identify pivotal players in this process. Approaches evaluating improved access of drugs to tumors such as nanoparticle formulations or condensate penetrants are furthermore being explored. An overview of these strategies is depicted in Figure 1. Early clinical trials have shown first signs of effectiveness for some of these approaches, but additional studies will be needed to translate this into future therapy options for PCa patients.

This review article focusses on novel approaches on how to block AR action, either directly by novel modalities or by addressing domains other than the LBD, or indirectly by blocking coregulators. Innovative ways to improve compound accessibility are also discussed. Literature was mainly searched in PubMed and Embase using as keywords “prostate cancer”, “androgen receptor”, “PROTAC”, “RIPTAC”, “degraders”, “splice variants”, “coactivators”, “pioneer factors”, “chromatin modifiers”, “nanoparticles”, “biomolecular condensates”. Details on clinical studies were obtained from ClinicalTrials.gov.

## 2. Targeting the AR with Degraders

### 2.1. SARDs

Small molecules that promote degradation of the AR, and in some instances of AR splice variants, via the E1/E2/E3 ubiquitin (Ub) proteasome cascade are collectively described as SARDs [42]. Early clinical efforts in this direction included the oral down-regulators AZD3514 and TAS3681. AZD3514 inhibits ligand-driven nuclear translocation and decreases AR protein levels primarily by reducing protein synthesis [43,44]. It was assessed in phase 1 clinical studies and showed moderate efficacy, but was not considered further due to gastrointestinal toxicity [45]. TAS3681 is a pure antagonist which down-regulates the levels of AR full-length and of the splice variant AR-V7, leading to strong growth inhibition of AR-V7-positive, enzalutamide-resistant PCa xenografts [46]. In a first phase 1 study carried out in heavily pretreated mCRPC patients, TAS3681 displayed some anti-tumor activity with a manageable safety profile [47]. While AZD3514 and TAS3681 demonstrated that pharmacologic reduction in AR and AR splice variant levels was feasible, they are not degraders. Conversely, the UT compounds, including UT-34, UT-69 and UT-155 which bind to the AR NTD, and in some cases also to the LBD, induce proteasome-mediated degradation of both AR full-length and AR-V7 [48]. UT-34 shows strong anti-tumor activity in human PCa models implanted into mice and rats [49]. It progressed into phase 1/2 clinical development under the name ONCT-534, but did not demonstrate a clinically meaningful improvement in patients [50]. Very recently, Z15 and its optimized derivative ZA5 were identified as SARDs which bind to the AR LBD and NTD, thereby resulting in degradation of AR and AR-V7 via the proteasome pathway [51]. Growth suppression was reported in resistant PCa models in vivo, but the pharmacokinetic properties of the compounds need to be improved [51]. These SARDs may overcome resistance mechanisms identified for anti-androgens such as AR point mutations and splice variants, and also AR overexpression, as reported for Z15 [52].

### 2.2. AR PROTACs

PROTACs are bi-functional compounds which bring together a target protein with a E3 Ub ligase to entice degradation of the target rather than just inhibiting its activity, so that the blockade may be more pronounced [53]. They are larger than SARDs as they engage two proteins so that oral bioavailability is often a challenge [54]. Nuclear and cytoplasmic proteins can both be addressed by PROTACs, with best efficiency achieved when the targeted protein and the recruited E3 ligase colocalize in the cell [55,56,57]. Most AR PROTACs described have a moiety binding in the LBD pocket and a moiety recruiting a Ub ligase, usually the Von-Hippel–Lindau (VHL) or the cereblon (CRBN) E3 ligase, ultimately leading to AR ubiquitylation and subsequent degradation by the proteasome [17,58,59,60].

Several AR PROTACs have been evaluated in clinical studies. Bavdegalutamide binds to the AR LBD and recruits the CRBN E3 ligase. It shows strong anti-tumor effects in several PCa models and was the first PROTAC to move into clinical trials [61]. Positive results and good tolerability were reported from a clinical phase study where bavdegalutamide combined with abiraterone acetate was evaluated in mCRPC patients [62]. The follow-up AR PROTAC luxdegalutamide showed promising results, as evidenced by declines in serum prostate-specific antigen (PSA) values, mainly in pretreated mCRPC patients with AR LBD mutations [63]. It is now assessed in mCRPC and in metastatic hormone-sensitive prostate cancer (mHSPC) patients in several clinical phase 2 studies, in combination with different agents. The PROTAC gridegalutamide (BMS-986365, CC-94676) binds to the AR LBD and recruits the CRBN E3 ligase, causing degradation of AR wild-type and clinically relevant mutants [64,65]. It has strong preclinical activity in several PCa models [64,65] and first clinical data revealed PSA reduction in mCRPC patients [66]. It is presently being evaluated in men with mCRPC in a clinical phase 3 study. The AR PROTAC HP518 is currently tested in a phase 1 dose-escalation study and responses in a subset of mCRPC patients have been reported [67]. AZD9750, an AR PROTAC binding to the AR LBD via a unique binding motif and recruiting the CRBN E3 ligase, shows strong preclinical anti-tumor effects [68]. A clinical phase 1/2 study to evaluate AZD9750 in metastatic PCa, including combination treatments with PARP inhibitors, has just been initiated [69]. GDC-2992 is an AR PROTAC that recruits the E3 ligase CRBN [70] currently being evaluated in a phase dose-escalation study in late-stage PCa patients [71]. Altogether, there are now several AR LBD PROTACs which are clinically being studied in mCRPC [17,42,53,58,59,60] and it will soon be known whether this approach represents an effective and durable strategy for patients with resistance to established therapies.

### 2.3. Novel PROTAC Approaches

Besides the identification of PROTACs binding to the AR LBD via different chemical scaffolds, efforts have been undertaken to target other AR domains [59,60]. The AR-V7 splice variant which is highly expressed in late-stage PCa is depleted of its LBD, leading to efforts towards targeting its NTD (see below). This however proved challenging, as the NTD is an intrinsically disordered region [72] but AR NTD-targeting PROTACs that recruit the E3 ligases VHL or CRBN have now been described [72]. AR and AR-V7 level reduction as well as in vitro and in vivo anti-tumor activity have been reported for several AR-NTD PROTACs, including BWA-522, NP18, ITRI-90 and ITRI-148 [73,74,75,76].

PROTACs have also been designed to target the AR DBD with MTX-23 representing one of the first examples [77]. It recruits the VHL E3 ligase and, like NTD-targeting PROTACs, induces degradation of both AR full-length and AR-V7. It reduces cell proliferation and tumor growth of PCa models that respond poorly to approved AR inhibitors [77]. Another example is the LYA914 PROTAC which binds to the AR DBD and recruits the CRBN E3 ligase, leading to significant in vitro and in vivo anti-tumor effects in different PCa models [78].

NTD- and DBD-targeting PROTACs represent a potentially promising strategy to address AR with point mutations in the LBD or splice variants depleted of the LBD which have been found in therapy-resistant PCa, but so far, the reported compounds are still in the preclinical stage, and their clinical value has to be demonstrated in additional studies.

Altogether PROTACs represent an attractive new strategy to efficiently address cancer targets. However, the recruitment of only a small subset of E3 ligases has been thoroughly studied until now, which may limit therapeutic translation, and there are now numerous ongoing efforts to extend the range [54,79]. Also, toxic effects due to degradation in healthy tissues are a potential challenge, motivating the exploration of pre-PROTACs, which are masked PROTACs that can be conditionally activated and are designed to limit target degradation until they reach the intended disease context [79]. There are also first studies showing that resistance to PROTACs may be acquired through mutations in components of the E3 ligase complex [80] or elevated expression of drug efflux pumps [81], and this needs to be considered in future research efforts.

## 3. Targeting the AR with RIPTACs

Small heterobifunctional molecules inducing the formation of complexes between a target protein abundantly expressed in tumors and a pan-essential effector protein (EP), thus resulting in diminished activity and tumor cell death, represent a novel strategy for precision cancer treatment [82,83]. This approach is particularly effective for nuclear target proteins, other advantages being the high selectivity of cell death and the possibility to address the protein at regions not necessarily directly involved in the oncogenic activity, thus increasing the space of potential new cancer targets [82,83].

The RIPTACs H001 and H003 engage the AR with a currently non-disclosed pan-essential protein, show anti-proliferative effects in AR-positive PCa cells and cause regression of a PCa model with amplified AR in vivo [84]. The RIPTAC HLD-0915 binds to the AR and to the bromodomain and extra-terminal motif (BET) family member bromodomain-containing protein 4 (BRD4), leading to regression of several preclinical xenograft PCa models resistant to AR pathway inhibitors [85]. A phase 1/2 study evaluating the oral HLD-0915 in patients with mCRPC has just been initiated, making this compound the first RIPTAC to enter clinical development and it should soon be known whether this modality represents a promising new way to treat PCa patients [86]. Determination of AR and BRD4 levels in treated patients may help to better understand their response to treatment.

## 4. Targeting AR Splice Variants

AR splice variants are rarely found in untreated, early PCa, but become enriched in castration-resistant disease [87,88]. More than 20 AR splice variants have been identified but only a subset of them has been thoroughly analyzed. The most studied splice variant is AR-V7 which lacks the LBD, is constitutively active, and is associated with elevated relapse risk and reduced overall survival in PCa patients, potentially due to its ability to drive a partly distinct transcriptional program linked to increased chromatin accessibility and non-canonical engagement [89]. Targeting the AR-V7 NTD has proven difficult despite intensive efforts, due to its unstructured organization, but several compounds with promising preclinical efficacy have been described and a few of them have later been evaluated in patients [72,90]. As an alternative future approach, factors involved in AR transcript splicing are being analyzed in detail and silencing studies have underlined their implication in PCa proliferation, bringing the hope that novel therapeutic strategies addressing this mechanism may be validated in the near future [88].

### 4.1. AR NTD Binders

AR splice variants are selectively found in castration-resistant PCa, with AR-V7 representing the most prominent one [89]. AR-V7 is constitutively active and regulates a distinct downstream transcription program which has been linked to elevated chromatin accessibility [91,92] and transcriptional repression [93,94]. Its impact on anti-androgen therapy resistance has been reported in many preclinical models [95]. A correlation between AR-V7 expression and increased CRPC progression has been evidenced in several reports, most recently in a review of seven clinical studies [96]. AR-V7 is mainly detected in circulating tumor cells (CTCs) but liquid biopsy and exosome analysis have now also been successfully used [95].

Several small molecules addressing the AR-V7 NTD have been described. The most advanced compounds act by binding to the AF-1 transactivation region, more specifically the TAU-1 and TAU-5 units which are essential for the transcriptional activity. Ralaniten acetate (EPI-506) was the first AR-NTD binder evaluated in mCRPC patients, but sufficient exposure could not be achieved in the phase 1 study, due to limited oral bioavailability [97]. Another AR-V7 NTD binder, masofaniten (EPI-7386), advanced to clinical phase 2, in combination with the AR inhibitor enzalutamide, also in mCRPC patients, but no benefit was observed based on PSA responses [72]. Masofaniten combined with enzalutamide and ADT was furthermore tested in mHSPC patients and this triplet regimen demonstrated preliminary efficacy and an acceptable safety profile; however, this program was subsequently discontinued [98]. Other AR NTD binders such as QW07, UT-143 and SC912, and also PROTACs (see above), have been shown to impair PCa growth in preclinical models, but they are still in early research phases [49,72,90].

### 4.2. AR Splicing Blockade

Trans- and cis-acting factors are both involved in aberrant splicing occurring during the progression of PCa [88]. A direct role of splicing factors in the abnormal expression of AR variants has been reported by several groups. U2AF65 and ASF/SF2 are splicing-enhancer binding proteins essential for the generation of AR-V7 mRNA [99]. The RNA-binding protein Src-associated substrate in mitosis of 68 kDa (SAM68) controls AR-V7 expression and function [100]. Another RNA-binding protein, PTB-associated splicing factor (PSF), promotes splicing of AR and its knock-down causes the reduction in AR and AR-V7 levels, and the subsequent growth reduction in PCa cells [101]. The X-linked RNA-binding motif protein RBMX is involved in the splicing control of AR transcripts and its knock-down leads to reduced levels of several AR splice variants [102]. The transformer 2 (TRA2) homologs TRA2A and TRA2B were recently identified as important regulators of AR pre-mRNA splicing and impairment of their activity is followed by reduced PCa proliferation [103]. Interestingly, a modulatory impact of AR on alternative splicing in PCa via regulation of the trans-acting factors epithelial splicing regulatory protein (ESRP) has been reported, and both ESRP1 and ESRP2 were found to be involved [104,105]. Another recent report shows that the serine/arginine-rich splicing factor 9 (SRSF9) binds to a sequence found in the 3′-untranslated region of the AR-V7 transcript and contributes to splicing [106]. AR-V7 splicing is also regulated by the YTH domain-containing protein 1 (YTHDC1), which binds to the AR-V7 pre-mRNA and recruits the splicing machinery [107]. Complementing these findings, an unbiased clustered regularly interspaced short palindromic repeats (CRISPR) screen where over 200 splicing factors were knocked-out led to the identification of the microfibril-associated protein 1 (MFAP1) and the CWC22 spliceosome-associated protein as essential for AR splice variant generation [108]. Their depletion led to impaired growth of several PCa cell lines [108].

These mechanistic insights have prompted preclinical efforts to interfere with AR variant generation by blocking aberrant splicing, for example using splice-switching antisense nucleotides directed against AR pre-mRNA or small molecules which reduce AR-V7 levels via targeting of key splicing regulators [87,109]. Splice-switching antisense oligonucleotides directed against cryptic splicing signals within the AR pre-mRNA, such as AON-ISE and the less potent AON-ESE, prevent AR-V7 mRNA synthesis without reducing full-length AR mRNA and down-regulate AR-V7 target genes, subsequently suppressing androgen-independent proliferation and inducing apoptosis in CRPC models [110]. Small-molecule splicing modulators provide a complementary approach. For instance, sulfonamide indisulam promotes the degradation of the splicing factor RNA-binding motif protein 39 (RBM39), thus leading to decreased AR-V7 mRNA and protein levels, and subsequently to strong growth inhibition of the AR-V7-positive VCaP PCa model [111]. In another study, indisulam and other splicing-targeting drugs were also found to reduce the growth of several PCa models, and this was linked to splicing alterations in transcripts encoding PCa-relevant proteins [112]. Thailanstatin is a ubiquitous splicing inhibitor that suppresses AR-V7 pre-mRNA expression by blocking the complex assembly of the splicing factors U2AF65 and SAP155, causing growth inhibition of AR-V7-positive cell lines and of the corresponding xenograft models [113]. Finally, blockade of the Cdc2-like kinase family perturbs the activity of SRSF9 which is essential for AR-V7 mRNA synthesis, thereby impairing proliferation of several PCa models [106]. Altogether these data support the concept of hindering the generation of AR splice variants by interfering with pre-mRNA maturation. It should, however, be noted that most compounds evaluated up to now broadly affect splicing rather than selectively modulate AR transcript processing, thus limiting the mechanistic conclusions and posing a challenge for clinical translation.

There are only a few clinical studies that evaluated the efficacy of AR splicing modulators in PCa. The antisense oligonucleotide EZN-4176 binds to exon 4 which encodes the AR hinge region, leading to down-regulation of AR and of several splice variants including AR-V7, and to growth inhibition of AR-positive tumor cell lines [114]. It was evaluated in an early-phase clinical trial in CRPC patients with progressing disease but showed only limited anti-tumor activity [115]. Other clinical studies evaluating splicing inhibitors in PCa patients dealt with compounds having a broad pharmacological spectrum [16]. As the factors involved in AR splicing are now better characterized there is hope that a more specific blockade can be achieved. However, these factors are typically involved in other splicing events, and the selective targeting of AR transcripts will remain challenging [108,109,112,113]. Addressing minor intron splicing which plays an important role in PCa progression may represent an innovative and promising approach. The small nuclear RNA U6atac has a crucial function in this process, and knock-down experiments showed anti-proliferative effects in PCa cell lines and patient-derived organoids [116].

## 5. AR-Coregulator Interactions

### 5.1. Overview

The AR interacts with several molecular chaperones in the cytoplasm, mainly the FK506-binding protein 51 (FKBP51), FKBP52 and heat-shock proteins, and following activation by androgen and nuclear translocation, with different cofactivators, pioneer factors and histone-modifying enzymes [6,117,118,119,120]. This leads to downstream activation and more rarely repression of target genes, followed by up- or down-regulation of the corresponding proteins, with however different levels of concordance, depending on the analysis method used [36,121,122,123,124,125]. A discordance between transcript and protein levels has also been reported during PCa progression [126]. In view of the pivotal role of partner factors on the full impact of AR function, efforts have been undertaken to identify and block those that have a major implication in tumor development and treatment resistance [6,117,118].

Genome-wide studies indicate that the AR interacts with multiple DNA-binding sites, including inter- and intragenic regions [127,128,129], and that these sites tend to accumulate mutations in PCa [130]. Regulatory enhancer and super-enhancer regions are usually co-occupied by diverse factors and form loops with distant promoters [129,131,132,133]. Detailed analysis to decipher AR cistrome changes, especially in a pathologic context, helped to further understand the impact of cofactor binding [134]. Reprogramming of the AR cistrome leading to alterations in downstream gene regulation is instrumental in the progression from normal prostate to primary tumors, and later to metastatic cancer and resistance to established therapies [128,135,136,137]. A reactivation of early prostate-specific development programs through AR binding at canonical sites has been reported [136]. The additional role of a non-canonical AR cistrome enriched in regulatory regions of genes coding for cell cycle players has been evidenced in PCa metastasis [138]. Another recent study reports that rewiring AR binding towards genomic sites bound by the Krüppel-like family (KLF) of TFs is followed by transition towards the neuroendocrine stage, and this is normally prevented by the enhancer of zeste 2 (EZH2), a chromatin modifier that catalyzes histone H3 lysine 27 methylation [139].

Clearly, the druggability of AR coregulators by small molecules needs to be considered when evaluating them as potential new drug targets for PCa. Here, chromatin modifiers and readers are probably better suited, in view of the tremendous progress achieved in recent years with regard to generation and availability of potent and selective inhibitors [140]. The challenge is generally higher for pioneer TFs, so that other members of the complex formed with the AR may be more appropriate for pharmacological blockade [141].

### 5.2. AR Coactivators

Early studies identified the p160 proteins, which interact in most cases with the AR NTD, as essential for downstream gene transcription [117,118]. They play major roles in both hormone-sensitive and castration-resistant PCa stages, as shown mainly by gene silencing experiments in preclinical models [117,118]. New technologies have allowed to much broaden our knowledge of AR-interacting proteins, which now amount to over 280 [19,118,142]. These coactivators bind to distinct AR regions and have diverse functions in cellular physiology and signaling. Detailed analyses of their roles helped to explain the tissue- and cell-specific complexity of androgen action [19,118,142,143]. Mapping of the domains involved in the interaction of the AR with associated proteins has been successful in several cases, but disrupting these contacts remains challenging [117,144,145]. The co-chaperone BAG-1L directly interacts with the AR NTD and LBD, thereby leading to N- and C-terminal domain interaction, recruitment of additional coactivators and stimulation of activity [146]. A compound preventing the interaction between the AR and BAG-1L reduces PCa cell proliferation, but only at a high concentration [147]. Another inhibitor, ASC-J9, prevents the association between the AR and associated coactivators, which is followed by AR destabilization and reduction in PCa cell proliferation [148]. In vivo efficacy was additionally reported for ASC-J9 in an AR-negative PCa xenograft model, implying a more complex mode of action [148]. ASC-J9 derivatives with predicted higher AR binding and enhanced cytotoxicity in PCa cell lines have subsequently been described [149].

### 5.3. Pioneer Transcription Modulators

The pioneer TFs forkhead box A1 (FOXA1), homeobox protein Hox-B13 (HOXB13) and GATA-binding factor 2 (GATA2), as well as the chromatin remodeling complex switch/sucrose non-fermentable (SWI/SNF) and the chromodomain helicase DNA-binding 1 (CHD1) protein were identified as major players in AR cistrome reprogramming [17,39,118,150,151,152]. Unbiased CRISPR-based screens performed to identify genes involved in AR function furthermore highlight the essential role of several of these factors [153,154].

FOXA1 induces chromatin opening and directly interacts with the AR, thus enabling binding to specific regulatory regions and downstream transcription activation [155,156]. Major changes in FOXA1 binding towards regulatory regions controlling genes involved in cell survival have been identified in patient tissues following treatment with enzalutamide [157]. A comprehensive analysis shows that FOXA1 gene alterations are found in nearly 35% of mCRPC patients and may lead to overexpression or to enhanced function [158]. In another study, FOXA1 coding mutations were reported in about 9% of primary PCa cases and in 13% of mCRPC cases [155]. Higher frequencies have been reported in tumors from Asian men, with 41% of localized prostate tumors found to have FOXA1 mutations [159]. The important role of FOXA1 post-translational modifications, including phosphorylation, acetylation and small Ub-like modifier attachment (SUMOylation), has furthermore been reported [156]. Compounds that covalently bind to a cysteine residue near the DNA-binding interface and alter the interaction of FOXA1 across the genome have been described [160]. Targeting of FOXA1 by small molecules remains a challenge so that the blockade of other interacting proteins, including epigenetic players, has been evaluated [156]. FOXA1 function can be reduced by upstream blockade of the acetyltransferase activity of CREB-binding protein (CBP)/p300, thereby attenuating AR signaling [161]. Bromodomain PHD finger transcription factor (BPTF) is part of a nucleosome-remodeling complex that stabilizes the AR-FOXA1 complex. This association can be disrupted by a compound binding to its bromodomain, leading to inhibition of PCa cell proliferation in a colony formation assay [162]. The levels of the related FOXA2 pioneer TF increase during PCa progression, which has been linked to stabilization following protein acetylation [126]. The collaborative impact of FOXA1 and FOXA2 in maintaining key transcriptional players involved in PCa progression has been reported and inhibitory compounds reducing chromatin occupancy and consequently clonogenic growth have been described [163].

The pivotal role of HOXB13 in androgen-controlled PCa growth has been reported by several groups in different models [164,165]. The function of this pioneer factor appears very selective for prostate tissue in the AR-positive and in the AR-negative context [166]. HOXB13 has an essential part in supporting AR-V7 binding to open chromatin, thus leading to oncogene expression [167]. Recent work indicates that both acetylation and phosphorylation of HOXB13 are crucial for oncogenic activity [133,165] and these post-translational modifications may offer opportunities for therapeutic intervention.

GATA2 controls the transcriptional activity of AR and AR splice variants and contributes to PCa aggressiveness and resistance to AR-directed therapies [154,168,169]. In silico screening allowed the identification of dilazep as a putative GATA2 inhibitor [170]. A diminished GATA2 chromatin recruitment and a reduction in PCa cell proliferation in vitro and in vivo were reported following dilazep application [170]. Another small molecule, K7174, decreases the binding of the AR-GATA2 complex to regulatory regions, resulting in reduced transcription and sensitization to an AR inhibitor [171]. Its full mode of action is, however, not completely elucidated.

CHD1 co-occupies enhancer regions also bound by the AR and its loss, which is common in PCa, redistributes the cistrome towards pro-oncogenic transcriptional programs [172]. CHD1 was identified as a synthetic lethal target in tumors deficient in phosphatase and tensin homolog (PTEN), and its inhibition by the small molecule UNC10142 impaired viability of PTEN-deficient PCa cells [173].

Importantly, CHD1 alterations often co-occur with mutations in the tumor suppressor speckle-type POZ protein (SPOP), a substrate adaptor for the cullin 3 Ub ligase complex that promotes AR ubiquitylation and proteasomal degradation [174]. SPOP is one of the most frequently mutated genes in PCa, in the range of 6–15%, mainly in the domain involved in substrate binding, and associated with a worse clinical outcome [175]. SPOP mutants also impair the ability of the Ub E3 ligase complex to degrade AR coactivators, thus leading to enhanced AR function [176]. Mutations in SPOP and copy-number loss of the paralog SPOPL have been reported in patients who respond exceptionally well to neo-adjuvant anti-androgen treatment [177]. In SPOP-mutated PCa, CHD1 loss enhances tumorigenesis by reprogramming the activity of the TF sterol regulatory element-binding protein 2 (SREBP2) to enhance cholesterol synthesis and intra-tumoral androgen biosynthesis, thereby fueling AR activity [174,176].

The ETS-related gene (ERG) encodes an important transcription regulator that increases SPOP expression, causing reduced AR signaling, but mutated SPOP represses ERG function, thereby increasing AR activity [178]. Allosteric surfaces involved in AR activation and cooperative interaction with ERG, and which contribute to resistance to AR-directed inhibitor treatment, have been identified [179].

Blocking the interaction or function of pioneer transcription regulators remains a challenge due to their nuclear localization and largely undruggable structure. Interestingly, a peptoid conjugate named MPC039 that modifies AR interactions with coregulatory proteins, compared to DHT-bound AR, has recently been shown to activate an anti-proliferative gene signature [180].

### 5.4. Chromatin Modifiers

Histone-modifying enzymes that interact with the AR are major players in cistrome reprogramming and treatment resistance [129,181,182]. Several compounds addressing these epigenetic targets are presently being tested in patients, often in combination with AR inhibitors, as outlined in several recent reviews [5,182,183,184].

Advanced examples include agents targeting the polycomb repressive complex 2 (PRC2) which methylates lysine 27 on histone 3, thus leading to repression of gene transcription [185]. The PRC2 complex plays a significant role in PCa and directly interacts with the AR [186]. Transcriptome analysis of patient-derived PCa xenograft models reveals that the PRC2 component EZH2, which is the enzyme responsible for histone 3 lysine 27 methylation and subsequent gene silencing, is one of the most up-regulated genes along the progression path [187]. Inhibitors of EZH2 or of embryonic ectoderm domain (EED), another PRC2 complex component, are being assessed in clinical studies. Single agents or combinations with AR inhibitors are being evaluated in hormone-sensitive and castration-resistant PCa [188]. Trial results from an ongoing phase 3 study testing the EZH2 inhibitor mevrometostat combined with enzalutamide in advanced PCa have very recently been presented [189]. Early clinical studies are additionally ongoing with other inhibitors of EZH2 or EED [190].

The histone 3 lysine 4 methyltransferase 2D (KMT2D) is overexpressed in PCa and recruits the FOXA1 pioneer factor to enhancer regions recognized by the AR, thereby promoting downstream gene activation [191]. Blockade of KMT2D function following inhibition of UTX, also known as lysine-specific demethylase 6A, leads to reduced AR signaling and proliferation inhibition [191].

The histone 3 lysine 36 methylase nuclear SET domain-containing protein 2 (NSD2) is an essential player in PCa progression and forms a complex with the AR and FOXA1 to occupy over 65% of the AR cistrome in prostate tumors [192,193]. A phase 1 clinical study evaluating the NSD2 inhibitor KTX-2001 as a single agent or in combination with darolutamide in mCRPC has just been initiated [194].

The lysine demethylases KDM1A and KDM7A remove mono- and dimethyl groups from lysine 4, or lysine 9 and lysine 27 in histone 3. These AR coregulators are involved in oncogenic transcription programs and display elevated expression levels in a subset of PCa [195,196,197]. Their inhibition reduces AR and AR target gene expression, and this has strong anti-proliferative and anti-invasive impacts on CRPC cell lines, especially when dual blockade is performed [197].

The histone acetyl transferases CBP and p300 are highly related, major AR cofactors that activate enhancers for downstream gene expression and promote PCa growth. Their involvement in the activation of the phosphoinositide 3-kinase (PI3K)/AKT and mitogen-activated protein kinase (MAPK) pathways, which crosstalks with AR signaling, has been evidenced in several studies [198,199]. In addition, a crucial role of CBP/p300 in DNA damage repair has been reported [200]. Several compounds that impair the acetyl transferase activity or the binding to the CBP/p300 bromodomain have shown effectiveness in PCa models [199]. Advanced compounds include inobrodib, a compound targeting the bromodomain and with anti-tumor effects in multiple PCa cell lines and xenografts [201]. First phase 1/2 data from mCRPC patients have been reported [201]. Pocenbrodib (FT-7051) also binds to the CBP/p300 bromodomain and is currently in a phase 1b/2a clinical trial to evaluate its impact as single agent or in combination with different agents in mCRPC patients [202]. Very recently, another CBP/p300 bromodomain blocking agent named CZL-077 was reported to have strong efficacy in several PCa xenograft models [203]. Other approaches include the CBPD-409 PROTAC which markedly degrades CBP/p300 in multiple cell lines [204]. This leads to reduction in acetylation marks in the N-terminal region of histone H2B which plays a crucial role in the activation of enhancers bound by the AR and CBP/p300 [204]. CBPD-409 has strong anti-proliferative activity in PCa models in vitro and in vivo, and synergy with AR inhibition was observed [204]. Preclinical data for a dual CBP/p300 and BET inhibitor named EP31670 have been reported [205]. This compound entered a phase 1 clinical study that includes patients with advanced PCa.

Histone acetylation marks are read by BET proteins via their bromodomains and an essential role of the BET protein family BRD4 in downstream AR signaling has been documented in several studies [132,206,207]. Intense efforts have led to the identification of several BET inhibitors that were subsequently evaluated in the clinic for different cancer indications, including PCa [184,208,209]. The BET inhibitor ZEN-3694, combined with enzalutamide, was evaluated in a phase 2 study in mCRPC and efficacy was reported in patients with low AR activity [210]. A limited impact was also reported for the BET inhibitor GS-5829 combined with enzalutamide in mCRPC patients [211]. More recently, the BET inhibitor BI 894999 was tested as single agent in an early clinical program enrolling patients with lymphoma or solid tumors, including a few mCRPC patients, but here also the efficacy was weak [212]. Additional ongoing clinical studies to evaluate BET inhibitors with different profiles in late-stage PCa will show whether this represents a viable therapy approach [184].

### 5.5. Challenges

As summarized above, addressing AR cofactor and pioneer transcription regulators remains a challenge, due to the undruggable structure of most of these targets. Concerning chromatin-modifying enzymes, potent and selective inhibitors are now described for many of them and several clinical studies in PCa patients are ongoing (Table 1). Overexpression in tumor and selective impact of gene silencing remain important criteria for target selection; however, side-effects due to the frequent pleiotropic function of these protein families will need to be monitored closely to ensure safety in treated patients.

## 6. Alternative Approaches for Improved Accessibility of Compounds

### 6.1. Nanoparticles

Nanoparticle formulation and delivery have the potential to increase solubility and permeability, and to achieve a controlled release of drugs. Nanoparticles containing the first-generation anti-androgen bicalutamide have been described and improved properties reported [213,214,215]. Concerning enzalutamide, insertion in a nanoparticle coated with the prostate-specific membrane antigen (PSMA) led to selective internalization into PCa cells and reduction in cell proliferation with diminished cytotoxicity [216]. In another study, enzalutamide and docetaxel were loaded into nanoparticles targeting PSMA and strong in vivo anti-tumor effects with limited toxicity observed for particles containing both drugs [217]. The AR signaling inhibitor abiraterone acetate has also been encapsulated in nanoparticles such as solid lipid nanoparticles, nanostructured lipid carriers and self-nanoemulsifying particles, leading in some instances to improved drug properties [218,219,220].

Importantly, nanoparticle approaches have also been explored to improve the pharmacokinetic profile of PROTACs, with the aim to increase tumor selectivity while keeping toxicity low [79]. First preclinical efficacy data for nanoparticles carrying the ARV-771 BET degrader in PCa models have just been published [221].

Nanoparticle formats were also evaluated to improve the performance of antisense oligonucleotides interfering with AR-V7 mRNA generation. The use of optimized AON-ESE nanoparticles led to superior delivery, strong AR-V7 knock-down and growth reduction in two androgen-independent PCa cell lines [222].

The nanoparticle delivery of immunotherapeutic treatments was analyzed in a multiphysics model including tumor shape and stromal density to better understand the resistance of prostate cancer to checkpoint inhibitors, and this may also be helpful for other nanoparticle treatments [223].

Altogether, these are all early studies and additional improvements, especially concerning scalability and safety, will be needed to enable translation into the clinic.

### 6.2. Targeting Biomolecular Condensates

Liquid–liquid phase separation where biomolecules condensate in membraneless compartments have important cellular functions, including gene expression regulation, and dysregulated condensates have been linked to transcriptional reprogramming and oncogenic processes [224,225,226]. Concerning the AR, biomolecular condensates that additionally include the mediator of RNA polymerase II transcription subunit 1 (MED1) and BRD4 were found to be enriched at super-enhancer regulatory regions in the genome [72,132]. This leads to stimulation of gene transcription and can be blocked by specific inhibitors [40,132,227]. A predominant player in the formation of AR condensates is the intrinsically disordered NTD region for which several binders have been described (see above). Intrinsically disordered regions have also been reported in the homeodomain TF OCT4 which interacts with the AR/FOXA1 complex in condensates located at shared DNA-binding sites [226]. Efforts directly addressing this aspect employing a phenotypic screen based on phase separation led to the identification of ET516, a compound that disrupts AR condensates [227]. This small molecule binds to the AR NTD, suppresses transcription and has in vivo anti-tumor effects in PCa models resistant to anti-androgens [227]. The AR NTD binder UT-143 mentioned above covalently binds to cysteine residues in the AF-1 domain and was also found to disturb condensate formation [228].

Clearly, more efforts will be needed to validate the concept of selectively addressing nuclear biomolecular condensates for PCa treatment and determine whether preclinical findings can be translated in patients. Also, safety aspects need to be closely monitored for this emerging approach. The recent fast-track designation by the Food and Drug Administration (FDA) of a condensate-modulating agent targeting the Wnt/β-catenin pathway in gastric cancer brings hope for future advances in this emerging research area in the near future [229].

## 7. Summary and Outlook

**Figure 2:**
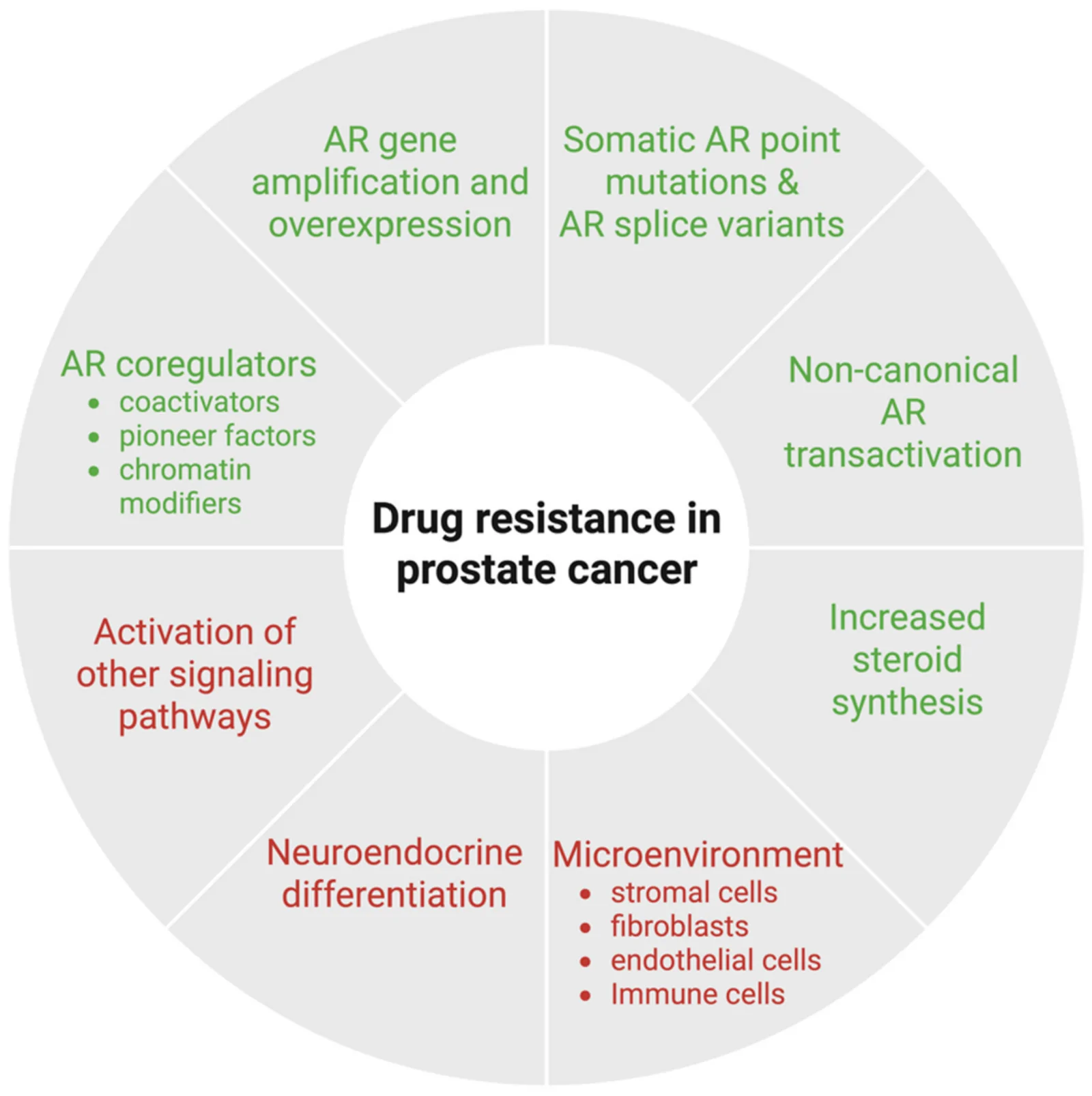
Overview of drug resistance mechanisms identified in PCa patients. Processes closely linked to AR signaling are indicated in green and AR-independent pathways in red. Created in BioRender: Dziubanska-Kusibab, P. (2026) https://BioRender.com/wsz04gb (accessed on 1 September 2026).

Compounds targeting the LBD to prevent androgen binding and thereby inhibiting AR function represent a well-established, essential treatment for PCa patients, and new approaches are currently being evaluated to further prolong efficacy while maintaining a good safety profile [4,16,42]. AR amplification, overexpression, mutations and splice variants are important mechanisms leading to drug resistance, so that new, creative strategies to overcome AR-driven resistance are of high interest [8,16,36,230,231,232] (Figure 2). Current research priorities include the evaluation of AR PROTACs and RIPTACs, binders of the AR NTD and DBD, and suppressors of AR splicing [58,60,82,83,85,87,88,233,234]. A complete blockade of AR function by protein degradation or attachment to an EP has the potential for a more sustained therapeutic impact in PCa patients. Multiple studies are now ongoing with AR PROTACs and it will soon be known whether this leads to clinical benefit. Concerning the targeted AR domains, despite numerous efforts, it has proven very difficult to find compounds with an impact comparable to competitive antagonists that bind into the DHT pocket. Selectively blocking AR splicing to prevent the generation of AR-V7 has proven difficult until now and will remain a challenge.

With regard to the efforts towards blocking essential factors that interact with the AR, including pioneer factors and chromatin modifiers (Figure 2), there are now promising results, particularly in combination treatment settings where AR signaling inhibitors are also given [235]. Addressing AR post-translational modifications such as phosphorylation, acetylation, methylation, ubiquitylation or SUMOylation may also represent a strategy to impair activity, but despite intensive efforts, no advanced compounds have been reported [236]. Concerning AR deubiquitylation, several Ub-specific proteases (USP) were reported to be involved in AR protein turnover and function, but only a few detailed studies with specific USP inhibitors have been published, so that the respective role of individual players still needs to be further elucidated [237]. AR SUMOylation is inhibited by the SUMO E1 inhibitor ML-792 leading to dramatic changes in AR binding to chromatin and transcriptional changes, but an essential role in PCa progression needs to be further clarified [238]. A different approach deals with long non-coding RNAs and there are accumulating data on their up-regulation in PCa resulting in stabilization of the AR and reduction in the impact of therapy, so that efforts are now ongoing to exploit this potential liability [239].

CRISPR-based screens performed to find mechanisms leading to sensitization or resistance to AR inhibitors bring hope that essential crosstalk pathways and drug targets will soon be identified [30,153,240]. Concerning advanced PCa, this also includes targets that do not directly interact with the AR pathway, which is particularly important given the fact that the number of late-stage patients developing treatment-induced neuroendocrine PCa where AR signaling is no longer the main disease driver is steadily increasing [23,241].

The constant increase in comprehensive genomic, epigenomic, transcriptomic and proteomic data from clinical samples will allow crucial progress in the PCa field, both for the identification of novel drug targets and plans on how to address them, and for the selection of biomarkers that reflect target engagement and predict the therapeutic effects. Emerging trends include combinations with epigenetic inhibitors and targeted radiotherapy [242]. Following many unsuccessful clinical studies with checkpoint inhibitors, there are now very new findings showing that a PSMA-targeted bispecific T-cell engager specifically activated in the tumor vicinity has beneficial effects in mCRPC patients [243]. All these advances will hopefully drive a new generation of more effective and personalized treatments for patients suffering from PCa.

## Data Availability

No new data were created or analyzed in this study. Data sharing is not applicable to this article.
